# Manufacturing and investigation of physical properties of polyacrylonitrile nanofibre composites with SiO_2_, TiO_2_ and Bi_2_O_3_ nanoparticles

**DOI:** 10.3762/bjnano.7.106

**Published:** 2016-08-05

**Authors:** Tomasz Tański, Wiktor Matysiak, Barbara Hajduk

**Affiliations:** 1 Department of Materials Processing Technology, Management and Technology in Materials, Institute of Engineering Materials and Biomaterials, Silesian University of Technology, Konarskiego 18a Str., 44-100 Gliwice, Poland; 2 Center for Nanotechnology, Silesian University of Technology, Konarskiego 18a Str., 44-100 Gliwice, Poland; 3 Centre of Polymer and Carbon Materials, Polish Academy of Sciences, 34 M. Curie-Sklodowska Str., 41-819 Zabrze, Poland

**Keywords:** ceramic nanoparticles, electrospinning methods, polyacrylonitrile, polymer composite nanofibres, spectroscopic ellipsometry

## Abstract

The aim of this study was to produce nanocomposite polymer fibres, consisting of a matrix of polyacrylonitrile (PAN) and a reinforcing phase in the form of SiO_2_/TiO_2_/Bi_2_O_3_ nanoparticles, by electrospinning the solution. The effect of the nanoparticles and the electrospinning process parameters on the morphology and physical properties of the obtained composite nanofibres was then examined. The morphology of the fibres and the dispersion of nanoparticles in their volume were examined using scanning electron microscopy (SEM). All of the physical properties, which included the band gap width, dielectric constant and refractive index, were tested and plotted against the concentration by weight of the used reinforcing phase, which was as follows: 0%, 4%, 8% and 12% for each type of nanoparticles. The width of the band gap was determined on the basis of the absorption spectra of radiation (UV–vis) and ellipsometry methods. Spectroscopic ellipsometry has been used in order to determine the dielectric constant, refractive index and the thickness of the obtained fibrous mats.

## Introduction

Over the last decade, there has been a noticeable development of materials from the group of polymer nanocomposites (PNCs), which are a combination of a polymer matrix with inorganic or hybrid nanoparticles. For the currently used types of inorganic fillers, inter alia, the following are included: SiO_2_ [1], metal oxides (TiO_2_, Al_2_O_3_, Bi_2_O_3_, Zn0, CaCO_3_) [2–6], metals (Au, Ag, Al, Fe) [7–9], SiC [10] and clays (saponite, montmorillonite, hectorite) [11–13]. In nanocomposites, which use inorganic materials at the nano-scale as reinforcement, there is a significant enhancement of properties with a much lower fraction of the reinforcing phase (≤10 wt %) compared to what is achievable using filler in the macroscale of the traditional composites. The used nanoparticles have a decisive effect on the properties of the produced composite. Therefore, by selecting the type of reinforcing phase and its amount, optical, magnetic, mechanical, thermal and tribological design properties of the obtained composite materials can be controlled.

The studies carried out hitherto have shown [14–17] that using SiO_2_ or TiO_2_ nanoparticles in a polymer matrix as the reinforcing phase (in the case of SiO_2_ even amounts as low as 1 wt % and without prior modification of the particle surface) significantly improves the mechanical properties and thermal strength of the resulting composite relative to the pure polymer. In addition, polymer composites (based on, inter alia, poly(ether ether ketone) (PEEK), poly(methyl methacrylate) (PMMA), poly(lactic acid) (PLA), poly(butylene succinate) (PBS), poly(3-hydroxybutyrate-*co*-3-hydroxyvalerate) (PHBV), poly(vinylidene difluoride) (PVBF), poly(vinylpyrrolidone) PVP, poly(acrylonitrile) (PAN)) reinforced with particles of SiO_2_, TiO_2_ and Bi_2_O_3_, particularly in thin layers, are very attractive because of their electrical, optical and photovoltaic properties. The properties of these oxides contribute to the fact that the materials obtained with their participation are successfully used for the construction of organic thin-layer transistors (OTFTs) [18], Schottky diodes [19] or sensors [20].

Currently, the most frequently used method for the preparation of polymer thin layers is the method of spin-coating, which uses centrifugal force to evenly distribute the polymer solution or melt on the selected substrate.

In recent years can be observed a significant increase of interest of composite nanofibres produced by using electrospinning from solutions of polymers based on polyacrylonitrile (PAN) and *N*,*N*-dimethylformamide (DMF) with ceramic nanoparticles of SiO_2_/TiO_2_/Bi2O_3_. PAN/SiO_2_ composite nanofibres are used as membranes in the production of air filters, gas absorbents and new types of lithium-ion batteries [21–24]. Studies on nanofibre composite mats of PAN/TiO_2_ have shown large photocatalytic efficiency under ultraviolet light and the potential use of these mats as catalyst in the decomposition of phenol, airborne aromatic compounds and methylene blue [25–28]. However, PAN/Bi_2_O_3_ composite nanofibres are used as intermediates to produce Bi_2_O_3_ ceramic nanofibres [29–30].

**Figure 1:**
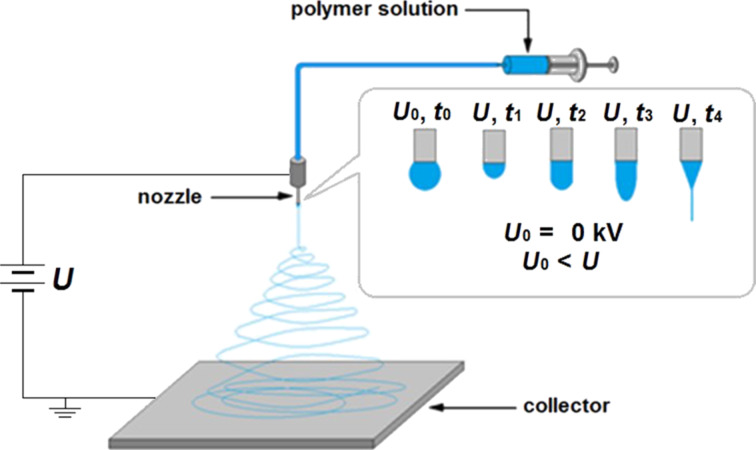
Schematic representation of electrospinning polymer nanofibres from solution.

Considering the above reports, the authors focus on obtaining thin composite layers of PAN nanofibres, reinforced with nanoparticles of SiO_2_, TiO_2_ and Bi_2_O_3_, through electrospinning from solution. It should be mentioned that the authors made an attempt to manufacture and investigate the properties of the PAN nanofibres reinforced with bismuth oxide nanoparticles, about which there is no literature data. Because of the good photocatalytic properties of Bi_2_O_3_ nanoparticles, nanofibres of PAN/Bi_2_O_3_ may be a more effective alternative to the widely described nanofibres of PAN/TiO_2_. This method is one of the most modern techniques for producing polymeric fibrous mats and micro/nano particles [31], and its application possibilities are extremely broad but not fully understood. In the process of electrospinning under the influence of a voltage between the nozzle and the collector, an electrostatic field is created, which causes induced charges on the surface of the spinning solution from the nozzle. Under the influence of the force of the electrostatic field, the drop in the nozzle opening is distorted and takes a conical shape (Taylor cone, Figure 1), accompanied by a movement of charges towards the grounded collector. The flow of the spinning liquid towards the collector is started when the critical current value is exceeded. It is stretched into a thin fibre under the field force. When the stream is reduced in diameter the ratio of the stream surface spray to its volume increases. The solvent evaporates and the spinning solution solidifies in the form of polymeric fibre, which is then "received" on the collector [32].

The aim of the work is to determine the influence of the type of used spinning mixture (polymer concentration, concentration and type of the reinforcement phase), and the electrospinning process parameters (the distance between the electrodes) on the morphology and the structure of the obtained composite nanofibres. Also the influence of nanoparticles and their concentration on the electrical and optical properties of the obtained composite nanofibres with the participation of fibrous layers are scrutinized by means of ellipsometry and UV–vis spectroscopy.

## Experimental

In order to prepare the spinning solution, the following were used: polyacrylonitrile (PAN purity 99%, *M*_w_ = 150 000 g/mol). The reinforcing phase was successively comprised of nanoparticles of SiO_2_ (99% purity, particle size of 12 nm), nanoparticles of TiO_2_ (purity 99%, particle size of 10–20 nm) and nanoparticles of Bi_2_O_3_ (purity 99%, particle size of 90–200 nm). The solvent was *N*,*N*-dimethylformamide (purity 99.8%). The final products were solutions of DMF/PAN (5 wt %)/nanoparticles (sequentially TiO_2_, Bi_2_O_3_, SiO_2_ at a concentration 0, 4, 8 and 12 wt %). In order to break the agglomerates of the reinforcing phase, a measured amount of nanoparticles was added to *N*,*N*-dimethylformamide, and the so-prepared solutions were subjected to sonification for 1 h, with automatic refill of the evaporating solvent. Immediately after sonication, a measured amount of polymer was added to the solution and subjected to mixing using a magnetic mixer for 24 h at room temperature. Immediately after mixing, the solution was placed in a sterile syringe as pumping device. Polymer nanofibres were obtained using electrospinning from solution using a FLOW - Nanotechnology Solutions Electrospinner 2.2.0-500 device. The fixed process parameters were: solution flow rate (0.7 mL/h), potential difference between the electrodes (19 kV), time of process (7 min). The distance between the electrodes was changed between 12.5 and 20.0 cm. Finally, four types of fibrous mats were obtained: (I) PAN polymer nanofibres, (II) PAN composite nanofibres reinforced with 4, 8 and 12 wt % TiO_2_ nanoparticles, (III) PAN composite nanofibres reinforced with 4, 8 and 12 wt % Bi_2_O nanoparticles and (IV) PAN composite nanofibres reinforced with 4, 8 and 12 wt % SiO_2_ nanoparticles. All of these groups of materials were obtained for two different distances between the electrodes of 12.5 and 20 cm, resulting in a total of 20 produced samples.

In order to identify the composition and morphology of the reinforcing phase, an X-ray analysis and a structural analysis of the applied ceramic nanopowders were carried out. In order to analyse the morphology and structure of the nanoparticles, an FEI Titan 80-300 high-resolution transmission electron microscope, which allows for imaging in the transmission and scanning mode, using bright and dark field (BF, DF), HAADF detector and an energy filter, in particular using analytical microscopy in the nanoareas in the STEM mode. The X-ray studies of the analysed materials were carried out on an PANalytical X’Pert Pro diffractometer. The measurement was performed in steps of 0.026 degrees and a step time of 30 s) in Bragg–Brentano geometry using the axis of the beam deflected of the detector PIXcel 3D. Co Kα radiation (λ = 0.17909 nm) supplied with a voltage of 40 kV at a current of glowing of 30 mA was used. For evaluating the obtained diffractograms, a dedicated database of PAN-ICSD files was used. The resulting polymer composite nanofibres were quantitatively and qualitatively analysed using EDX microanalysis and surface topography imaging using a Zeiss Supra 35 SEM, with a Trident XM4 X-ray spectrometer supplied by EDAX. Based on the SEM micrographs (50000× magnification), the diameters of the randomly selected nanofibres were measured using the DigitalMicrograph software, and then their mean value and the chemical composition based on EDX spectra were specified. In order to study the optical properties of the obtained fibrous polymer and composite layers, the nanofibres were applied on a silicon substrate, and then examined by means of ellipsometry. The study involved selected fibres produced with an electrode distance of *d*_1_ = 20 cm, due to the fact that these fibrous mats had a much lower average thickness than those produced at *d*_2_ = 12.5 cm. This significantly influenced the quality of the measurements of the refractive index *n*, the extinction coefficient *k*, and the real and the imaginary part of the dielectric constant ε. Ellipsometry measurements were carried out using a Sentech SE 850 E ellipsometer in the wavelength range of 240–2500 nm. The device was controlled by using the SpectraRay 3 software. The measurements of ψ and Δ were carried out under a constant falling light angle of 70°. The measurements of the absorbance of the obtained materials, from which the value of the data bandgap width was specified, were carried out using a Thermo-Scientific UV–vis Evolution 220 spectrophotometer. During the studies, a light beam with a wavelength in the range of 190–1100 nm fell perpendicularly on the sample. The value of the thickness of the obtained polymer and the composite mats was less than 1 μm, which guarantees that the results of the ellipsometry and UV–vis analyses are characterised by high accuracy and low error.

## Results and Discussion

### 1 Structural analysis of the nanoparticles

**Figure 2:**
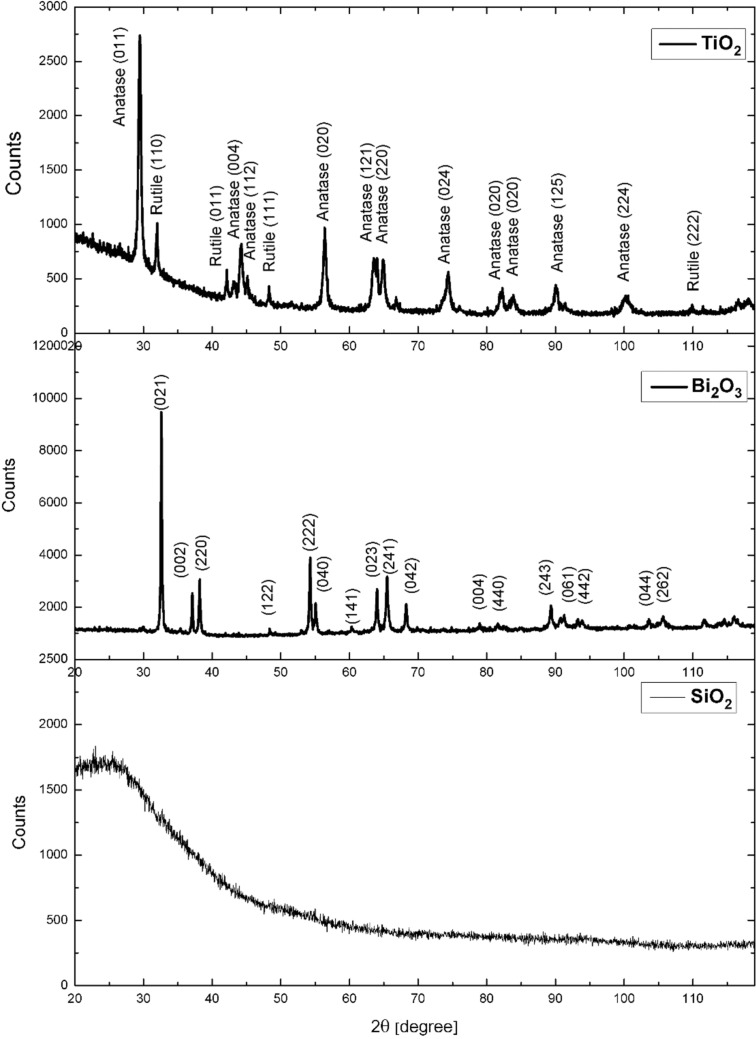
XRD spectra of ceramic nanoparticles.

The diffractogram of the TiO_2_ nanoparticles (Figure 2) shows diffraction lines characteristic for a tetragonal rutile (space group *P*4_2_/*mnm*, 98-008-2083 card) and nanocrystalline anatase (space group *I*4_1_/*amd*, 98-015-4604 card), were confirmed. For Bi_2_O_3_ nanoparticles_,_ the diffraction lines indicated the tetragonal structure of β-Bi_2_O_3_ (space group *P*−42_1_*c*, 98-005-2732 card). In the case of SiO_2_ nanoparticles, a wide and blurred diffraction line was observed, the so-called widening of the liquid, which indicates an amorphous crystal structure (Figure 2). The angular position of the diffraction line was, according to data contained in the ICDD JCPDS database, that of amorphous SiO_2_.

**Figure 3:**
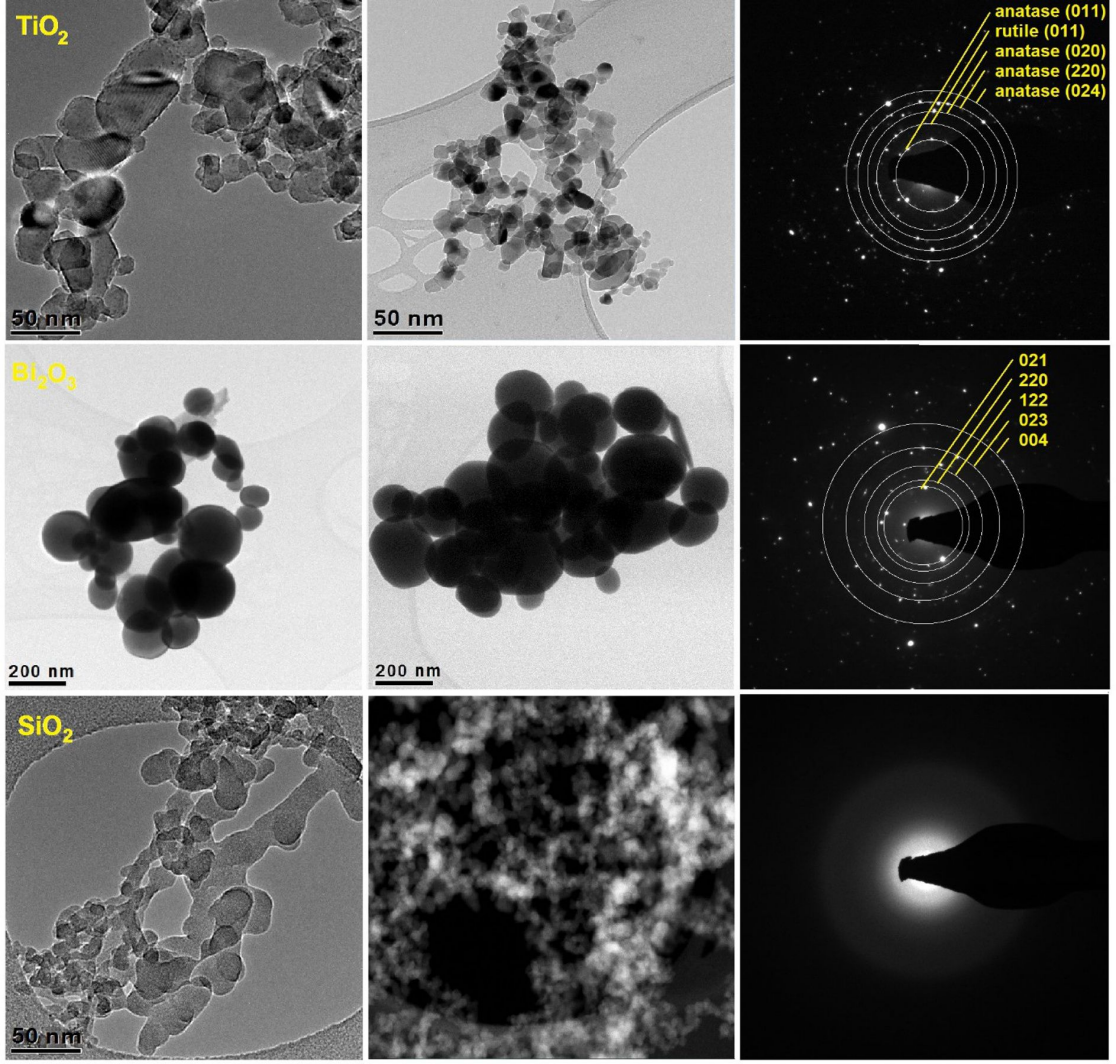
TEM images of the studied nanoparticles with diffraction images from single particles obtained using analytical electron microscopy in nanoareas in the STEM mode and the resolved diffraction patterns for TiO_2_ and Bi_2_O_3_ nanoparticles.

Figure 3 shows TEM images of the studied nanopowders taken in bright field and dark field mode and using an HAADF detector. The results of the diffraction studies, obtained using analytical electron microscopy in nanoareas in the STEM mode, confirmed the phase composition and the crystalline structure from the earlier X-ray study. The diffraction pattern obtained for TiO_2_ particles shows the (011), (020), (220) and (024) diffraction reflections corresponding to nanocrystal anatas, and (011) reflections of tetragonal rutile. In the case of Bi_2_O_3_ nanoparticles, diffraction occurred at the (021), (220), (222), (241), (243) planes of tetragonal β-Bi_2_O_3_. The fuzzy circle obtained for SiO_2_ nanoparticles confirms its amorphous form. An analysis of the TEM images, carried out for all used ceramic powders, has confirmed, in the case of each of these, the spherical morphology and variation of the particle diameters of the used nanofiller. The largest particle diameters in the range from 80 to 200 nm belonged to the bismuth oxide powder (Figure 3). For titanium oxide the obtained particle diameters ranged from 18 to 50 nm. The lowest measured diameter values were registered for silicon oxide nanoparticles, the particle sizes of which were in the range from 8 to 25 nm. The microscopic study for the analysis of TEM images of particles and the analysis of SEM images of the fibrous surface topography of the composite mats allowed us to determine the influence of the diameter of the applied ceramic nanoparticles on the structure and the morphology of the composite nanofibres (section 2), and the electrical as well as the optical properties derived from their division of the fibrous composite mats (section 3).

### 2 Analysis of the morphology of the nanofibres

**Figure 4:**
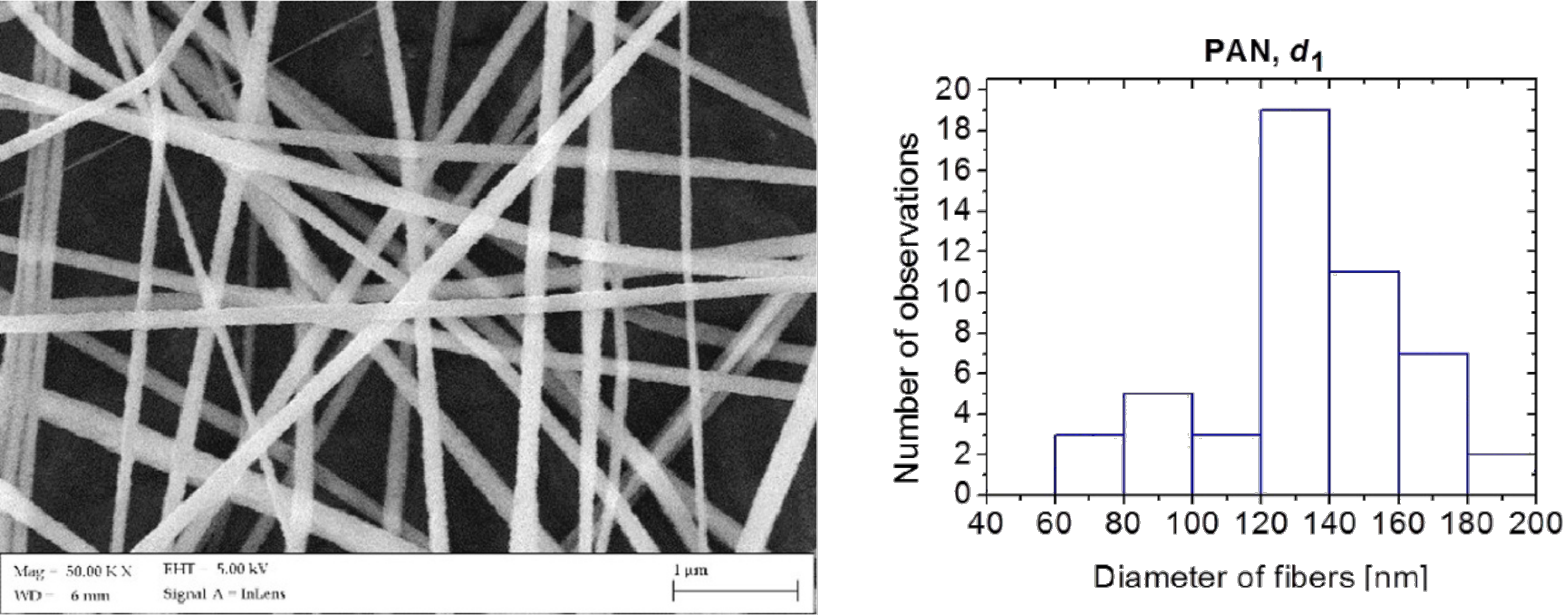
An SEM image of the morphology of PAN nanofibres taken at an electrode distance of *d*_1_ = 20 cm and a histogram showing the distribution of the measured diameters.

In order to analyse the morphology and the structure of the obtained polymer nanofibres used to produce the composite fibrous materials, surface topography imaging was applied using the scanning electron microscope. An analysis of the morphology and the structure of PAN nanofibres, obtained from a solution of PAN/DMF with a concentration of 5 wt %, with the distance between the electrodes equal to 20 cm, showed that these fibres are devoid of structural defects and are characterised by a constant diameter along their entire length (Figure 4). The fifty-time measurement of the diameters of the obtained PAN fibres showed that the measured diameters ranged from 60 to 200 nm, with the most common diameter values contained in the range of 120 to 140 nm, which represented 38% of all measured values for the diameter of the sample (histogram in Figure 4).

**Figure 5:**
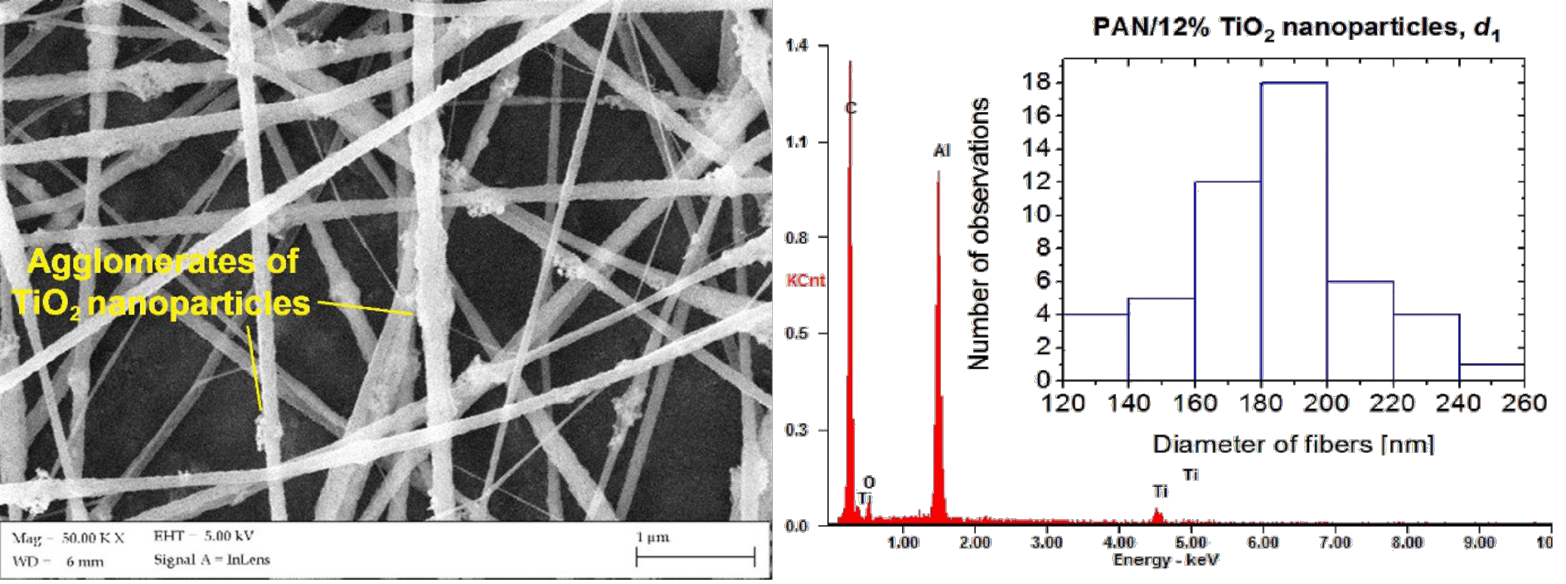
An SEM image of the morphology of PAN/TiO_2_ nanofibres with 12 wt % TiO_2_ produced at a distance of *d*_1_ = 20 cm, with the indicated nanoparticle agglomerates on the surface of the fibres, the EDX spectrum of the obtained fibres and a histogram showing the distribution of the measured diameter.

The SEM images the PAN nanofibres reinforced with TiO_2_ nanoparticles showed that the obtained composite fibres, unlike PAN fibres without reinforcement, are characterised by numerous structural defects, usually occurring on their surface (Figure 5). In addition, with an increase in the concentration of the used reinforcing phases in the produced nanocomposites an increase of defects and an increased heterogeneity of the fibre morphology was identified. X-ray microanalysis of the microareas showed that the observed defects in the structure of the nanofibres in most of the analysed cases were local conglomerations of TiO_2_ nanoparticles. This probably follows from strong interactions of the ceramic nanoparticles. The largest number of defects in the structure of composite PAN/TiO_2_ nanofibres was observed for samples with the highest concentration of the strengthening phase, produced with a 20 cm distance between the nozzle and the collector. The measured diameter values of the composite nanofibres reinforced with titanium oxide included values in the range from 120 to 260 nm, with the largest group, representing 36% of all of the fibres of this sample, having diameters of 180–200 nm (histogram in Figure 5).

**Figure 6:**
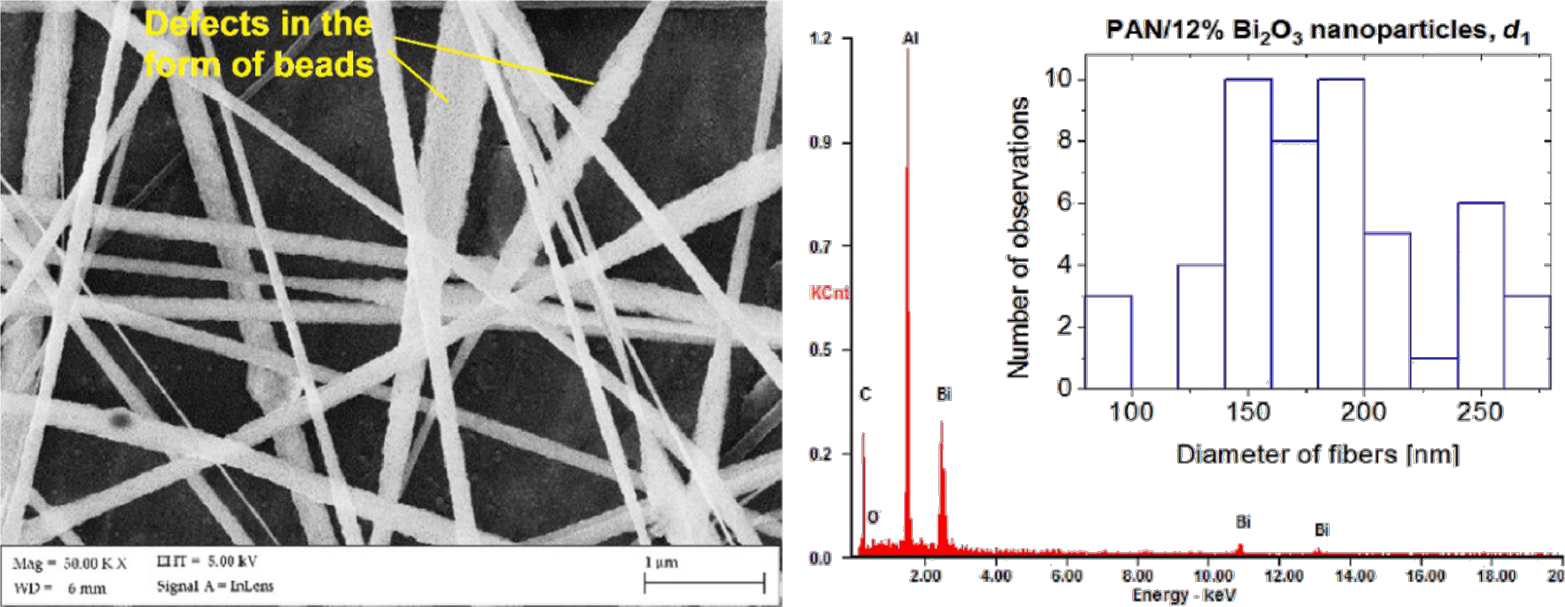
An SEM image of the morphology of PAN/Bi_2_O_3_ particles with 12 wt % nanoparticles produced at *d*_1_ = 20 cm, with the indicated agglomerates of nanoparticles on the fibre surface, the EDX spectrum of the obtained fibres and a histogram showing the distribution of the measured diameters.

The morphology of the nanocomposites fibres reinforced with 4 and 8 wt % Bi_2_O_3_ was characterised by a uniform cross section along the entire length. The morphology studies of composite PAN/Bi_2_O_3_ nanofibres and the EDX analysis showed no defects in the form of agglomerates of the strengthening phase. However, with an increase of the concentration of Bi_2_O_3_ nanoparticles in the polymer to 12 wt %, the formation of spindled beads can be seen (Figure 6), which are structural defects of composite nanofibres, produced at a distance of 20 cm between the nozzle and the collector. The fifty-time diameter measurement showed that the diameters of the fibres are in the range from 80 to 280 nm, and the fibre diameters ranging from 140 to 160 nm and from 180 to 200 nm are the largest (40%) group of the nanofibres (histogram in Figure 6). Defects in the form of spindled beads and of spherical shapes belong to the most common structural defects of nanofibres produced with the electrospinning method [33].

**Figure 7:**
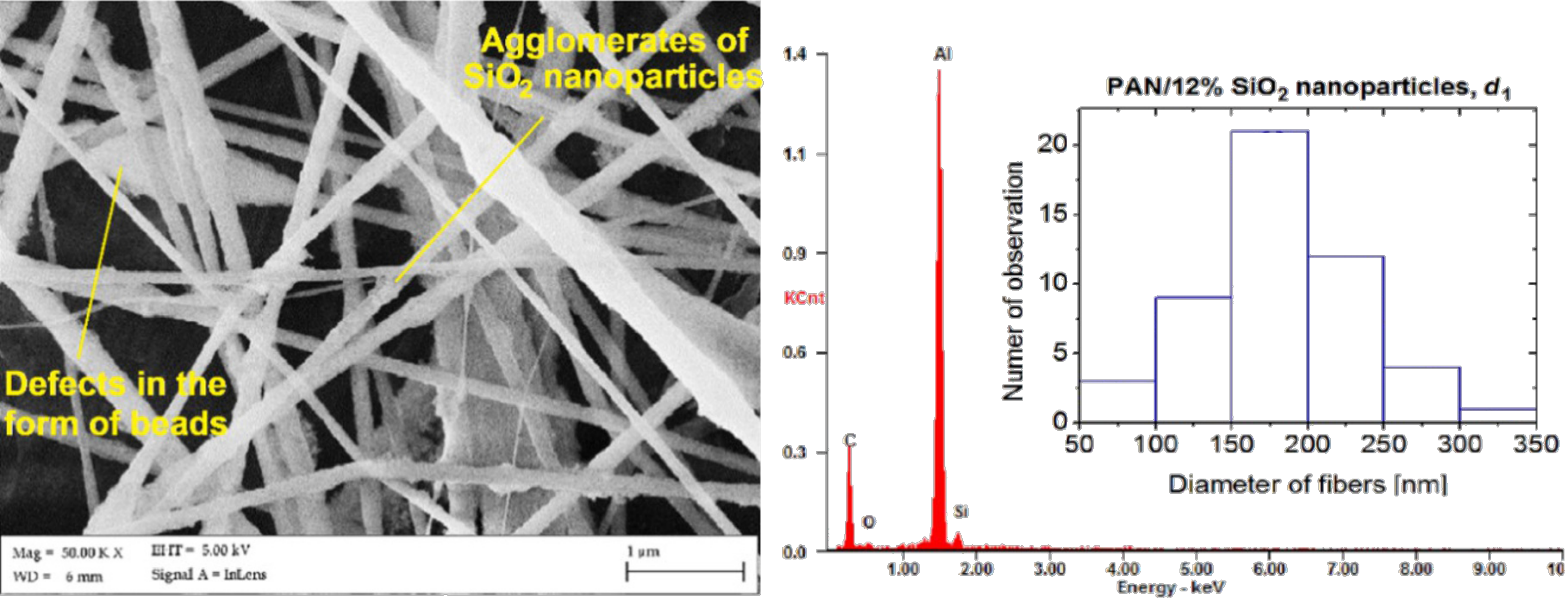
An SEM image of the morphology of PAN/SiO_2_ nanofibres with 12 wt % nanoparticles produced at *d*_1_ = 20 cm, with the indicated agglomerates of nanoparticles on the surface of the fibres, the EDX spectrum of the obtained fibres and a histogram showing the distribution of the measured diameters.

The comparison of the morphology of PAN/Bi_2_O_3_ nanofibres with the results presented in [31] shows a significant influence of the used polymer concentration in the spinning solution on the morphology of the obtained material. By using a solution made from the same materials, using constant electrospinning parameters (voltage of 15 kV) and doubling the mass of PAN to a 10 wt % solution, the composite PAN/Bi_2_O_3_ microparticles can be obtained. An analysis of SEM images of the topography of the surface of fibrous mats, carried out with composite PAN nanofibres reinforced with nanoparticles of SiO_2_, revealed the presence of a significant number of defects in the structure of the obtained nanofibres (Figure 7). These fibres are characterised by the greatest number of defects in comparison to composite nanofibres reinforced with TiO_2_ and Bi_2_O_3_. For all of the applied concentrations of the strengthening phase and the distances between the electrodes, in the case of composite nanofibres of PAN/SiO_2_, both local conglomerates of nanoparticles on the surface of the fibres were observed, which was confirmed through an analysis of the EDX spectra (Figure 7) and the defects in the form of spindled beads, which can be assessed by comparing the obtained composite nanofibres with nanofibres obtained from a pure polymer. The formation of numerous defects in the structure of the composite nanofibres reinforced with SiO_2_ probably results, just like in the case of the TiO_2_ powder, from the presence of strong interparticle interactions occurring between particles of the used strengthening phase, which, in turn, lead to the formation of visible agglomerates of nanoparticles. Based on the SEM analysis of the surface topography of the produced fibrous mats and TEM analysis of the particle size of the used nanopowders, it can be concluded that the formation of defects in the structure of the obtained nanofibres, probably resulting from the tendency to form agglomerates of the used strengthening phase, is associated with the size of the nanoparticles. Using particles of smaller diameters results in an increased number of defects in the structure of the obtained nanofibres. The tendency to connect nanoparticles in the agglomerates can be explained by the fact that smaller particles of SiO_2_, the average value of which was equal to a diameter of 17 nm, are characterised by a much larger area of approx. two times the area of TiO_2_ particles and more than eight times larger than the area of Bi_2_O_3_ particles, as a result of which the forces of adhesion per mass unit, responsible for the ability to connect to the reinforcing phase in the agglomerates, are larger. In addition, the analysis of the diameters of the obtained composite PAN nanofibres reinforced with silicon dioxide nanoparticles showed that these fibres are characterised by the greatest variety of diameter values in the range from 50 to 350 nm, compared to the other produced fibres (histogram in Figure 7).

#### 2.1 Influence of the distance between the electrodes and the mass concentration of nanoparticles on the diameter of the fibres

**Figure 8:**
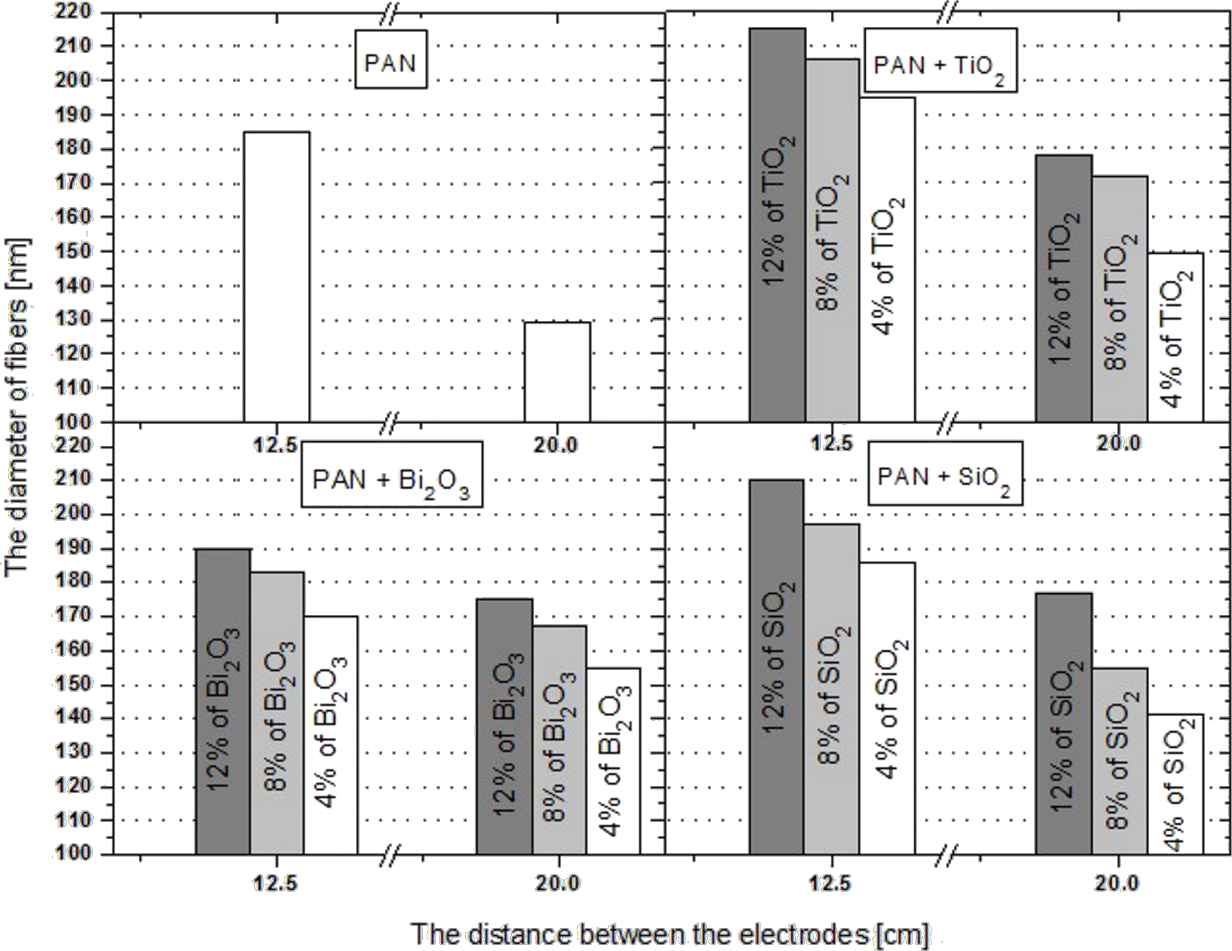
Calculated diameters of nanofibres as a function of the distance between the nozzle and the collector for all produced samples.

The most frequently used distances between the nozzle and the collector, used during the electrospinning of composite PAN nanofibres reinforced with ceramic nanoparticles while applying a potential difference in the range of 15–21 kV, are between 10 and 15 cm. The composite nanoparticles obtained under these conditions have diameters of 300 and 800 nm [34–36]. In order to better understand the relationship between the electrode distance and the diameter of the obtained fibres, we used two different distances between the nozzle and the collector, namely 12.5 and 20.0 cm. The diameter of the obtained fibres was determined by SEM analysis (Figure 8).

In the case of the smaller distance, the diameter of the fibres as a function of the mass concentration of TiO_2_ takes a linear form, and the diameter of the obtained fibres increases, on average, 10 nm for every 4 wt % of increase of the strengthening phase. When using nanoparticles of Bi_2_O_3_ as the reinforcing phase, the increase in the diameter of the nanofibres is approximately linear (Figure 8), with an increase of approx. 10 nm for every 4 wt % of Bi_2_O_3_, comparable to the titanium dioxide case. For a shorter distance between the electrodes, the diameter of the obtained nanofibres increases from 170 nm (4 wt % Bi_2_O_3_) to 190 nm (12 wt % Bi_2_O_3_). The use of a larger distance between the nozzle and the collector lead to an increase of the diameters from 175 nm (4 wt % Bi_2_O_3_) through 183 nm (8 wt % Bi_2_O_3_) up to 195 nm (12 wt % Bi_2_O_3_). Also in the case of composite fibres of PAN/SiO_2_, an increase in their diameter accompanying an increase of weight percentage was observed. Raising the mass concentration of SiO_2_ from 4% to 12% (at an electrode distance of 12.5 cm) resulted in an increase in the diameter, on average, of approx. 12 nm every 4 wt % (from 186 to 210 nm). When a distance of 20 cm was used, the diameter increased from 141 nm (4 wt % SiO_2_) through 155 nm (8 wt % SiO_2_) up to 177 nm (12 wt % SiO_2_), on average, 13 nm every 4 wt %. The observation revealed that using a reinforcing phase significantly influenced the diameter of the composite nanofibres. This is confirmed by the fact that for both electrode distances, an increase in the reinforcing phase is accompanied by an increase in the diameter of the obtained composite fibres.

The fact that a smaller electrode distance leads to an increase in the thickness of the fibres can probably be explained by less force acting on the induced electrical loads in the spinning solution, when the distance between the electrodes is equal to 12.5 cm, compared to the force that influences the induced loads at a distance of 20 cm. The electrostatic force resulting in stretching of the polymer stream is inversely proportional to the distance between the charges gathering on the surface of the collector and the charges induced in the solution that drop from the nozzle. Using a greater distance between the nozzle and the collector reduces the value of the force acting on the induced loads. But the actual distance covered by the polymer stream is much greater. This is associated with the electrostatic force acting for much longer time. This longer action of the electrostatic force on the induced loads leads to a longer time of stretching of the polymer and hence to the formation of fibres with smaller diameters.

The obtained composite PAN/SiO_2_ nanofibres, while maintaining a 20-cm distance between the electrodes, are characterised by an approx. 30% smaller diameter than the ones presented in [23], which is associated with a much larger surface. This fact shows that the obtained composite mats, reinforced with silicon oxide, are a promising starting material that can be used to produce carbon anodes, which are used in lithium-ion batteries, after their subsequent treatment by carbonisation and chemical removal of the reinforcing phases in order to increase the porosity of such obtained carbon fibre.

As it was demonstrated by Chureerat Prahsarn and collaborators [34], the polymer nanofibres of PAN are a very good medium for TiO_2_ nanoparticles with photovoltaic properties, providing the particles with stable embedding, while preserving the large contact area of the catalyst with the reagents, thus resulting in an increase of the effectiveness of photodecomposition processes. At an electrode distance of 20 cm we obtained composite PAN/TiO_2_ nanofibres with diameters smaller by approx. 40%, than to those obtained in [34], and by 78% smaller than the diameters of composite nanofibres obtained by Ji Sun Im, Min Il Kim and Young-Seak Lee [36]. This leads to a significant increase in the specific surface area of the composite mats related to receiving more efficient photocatalyst materials. It is anticipated that the Bi_2_O_3_-reinforced composite PAN nanofibres produced by the authors hereof, due to their photocatalyst properties [37], can provide a more effective alternative to the previously produced PAN/TiO_2_ nanofibres.

In addition, in the a stage of the study, it is planned to examine the effects even larger distances between the electrodes while maintaining the same parameters of solutions and the same flow rate.

### 3 Analysis of the physical properties of the obtained layers

#### 3.1 Ellipsometry studies of the produced layers

An assessment of the thickness of the produced fibrous layers was carried out using ellipsometric analysis (Table 1). The polymer mats of PAN reinforced with nanoparticles of Bi_2_O_3_ have the highest average fibre layer of 382 nm. In the case of using TiO_2_ as the reinforcing phase, this thickness was 296 nm and was approx. 10 nm larger than the thickness of the obtained layer of PAN nanofibres. The smallest thickness was observed after using the SiO_2_ nanopowder, and it was 262 nm, smaller than the thickness of the layers produced from pure polyacrylonitrile.

**Table 1:** The results of ellipsometric measurements of the thickness of the obtained fibrous layers produced at a distance of 20 cm between the electrodes.

sample	layer thickness [nm]

PAN	286
PAN/4 wt % TiO_2_	299
PAN/8 wt % TiO_2_	291
PAN/12 wt % TiO_2_	298
PAN/4 wt % Bi_2_O_3_	383
PAN/8 wt % Bi_2_O_3_	380
PAN/12 wt % Bi_2_O_3_	382
PAN/4 wt % SiO_2_	263
PAN/8 wt % SiO_2_	272
PAN/12 wt % SiO_2_	250

**Figure 9:**
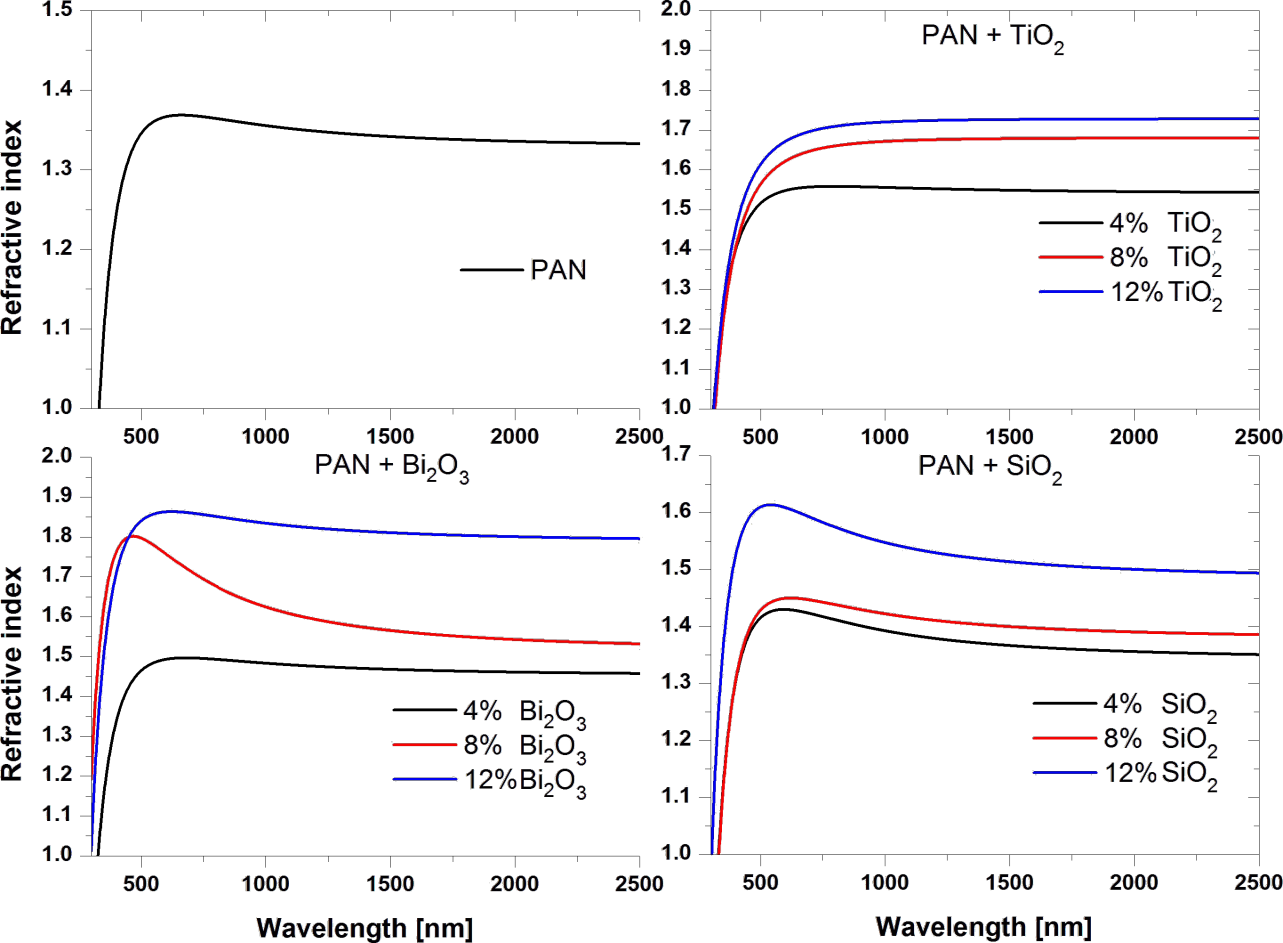
Refractive index of the produced fibrous layers as a function of the wavelength.

Ellipsometric analysis was used to measure the values of the refractive index (*n*) of the samples as a function of the wavelength in the range of 300–2500 nm (Figure 9).

The lowest value of refractive index of 1.34 was measured for the layer made from PAN fibres (Table 2), whereby the value stated by the producer Sigma Aldrich is 1.51. For all of the layers of the PAN/nanoparticles composite the obtained refractive index values were larger than the refractive index obtained for fibres of non-doped polymer. Adding 4 wt % of TiO_2_ nanoparticles to the polymer resulted in an increase of the refractive index to 1.55. A further increase of the TiO_2_ content caused an increase of *n*. For 8 wt % concentration the obtained value of *n* was 1.68, while for the maximum concentration of nanoparticles it was 1.73. Adding nanoparticles of 4 wt % Bi_2_O_3_ resulted in an increase of the refractive index to a value of 1.46. Adding more Bi_2_O_3_ (8 wt %) resulted in a further increase. The layer with the maximum concentration of of Bi_2_O_3_ (12 wt %) exhibited the largest achieved refractive index, *n* = 1.80. The lowest increase in the refractive index was obtained using nanoparticles of SiO_2_ as the reinforcing phase. Using 4 wt % silicon dioxide nanoparticles, the the refractive index increased by 0.02 in relation to the designated coefficient for pure polymer, while for 8 wt % SiO_2_ *n* was 1.39. The largest value of the refractive index in the case of nanoparticles of SiO_2_, was achieved for 12 wt % and was equal to 1.50.

**Table 2:** The results of ellipsometric measurements of the refractive index of the obtained fibrous layers produced at a distance of 20 cm between the electrodes (the values are provided for a wavelengths of 1800 nm).

sample	refractive index

PAN	1.34
PAN/4 wt % TiO_2_	1.55
PAN/8 wt % TiO_2_	1.68
PAN/12 wt % TiO_2_	1.73
PAN/4 wt % Bi_2_O_3_	1.46
PAN/8 wt % Bi_2_O_3_	1.55
PAN/12 wt % Bi_2_O_3_	1.80
PAN/4 wt % SiO_2_	1.36
PAN/8 wt % SiO_2_	1.39
PAN/12 wt % SiO_2_	1.50

For all of the groups of the produced composites an increase of the refractive index with increasing mass concentration of the reinforcing phase was observed. The lowest increase of *n*, obtained for composite PAN nanofibres reinforced with nanoparticles of SiO_2_, is due to the relatively low refractive index of SiO_2_, which is about 1.45 for visible light. The used polymer and the SiO_2_ particles have a similar *n*. This means the mechanism of interaction of electromagnetic radiation with these materials is similar, as evidenced by the results of studies of the optical properties. The refractive index values obtained for composites of PAN reinforced with titanium oxide and bismuth oxide are similar. This is due to the relatively small difference between the refractive index, for TiO_2_ approx. 2.6 and for Bi_2_O_3_ approx. 2.5 respectively. This suggests a much larger *n* of the composites produced with these nanoparticles compared to materials reinforced with SiO_2._

The dielectric transmittance as a function of the frequency of the incident radiation on the sample can be presented in the following form [38]:

[1]
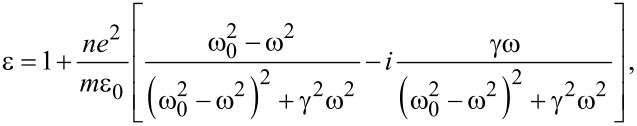


where *n* is the concentration of the atoms in the sample, *e* and *m* are charge and mass of the electron, ε_0_ is the electrical transmittance of vacuum, γ is the damping coefficient, ω_0_ and ω are, respectively, the frequency of the electron and the electromagnetic radiation. The above equation shows that the dielectric constant is a complex value, but its real and imaginary parts can be written as:

[2]
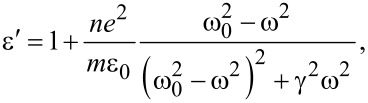


[3]
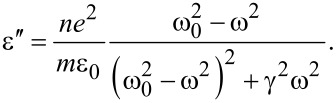


Using the relation connecting the refractive index and the dielectric constant, *n* = ε^1/2^, for expressing the real and imaginary part of the optical transmission, takes the form of [39]:

[4]
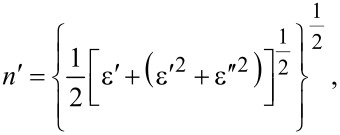


[5]
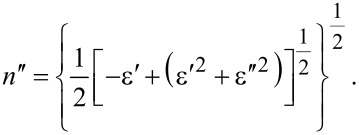


**Figure 10:**
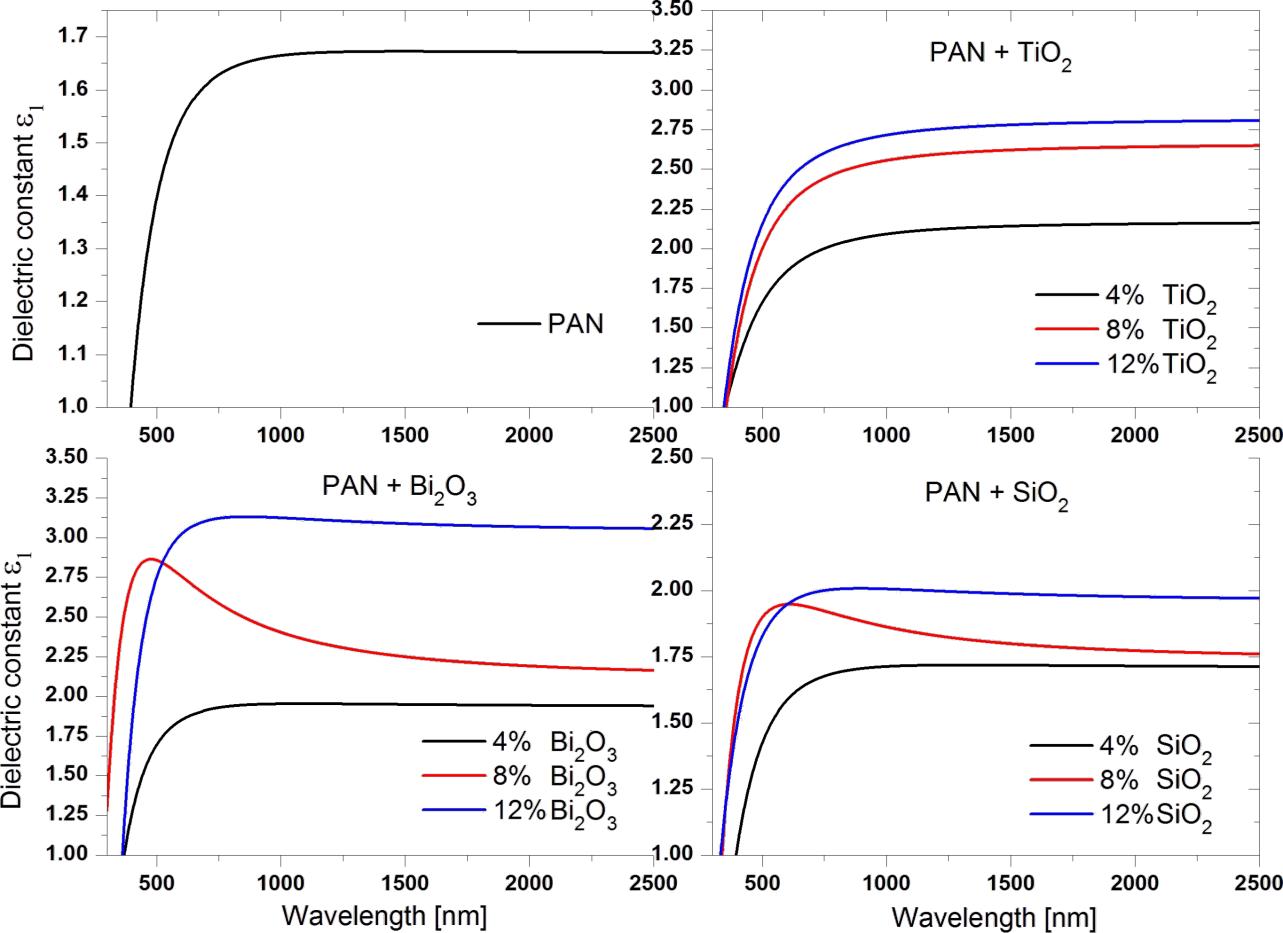
The dependence of dielectric transmittance as a function of the wavelength for the produced fibrous layers.

The above equations show that the higher the refractive index the higher the dielectric transmittance. The designated values ε (Table 3) were provided for a wavelength of 1800 nm. They show, similar the refractive index, a stabilisation of the dielectric constant for both pure polymer fibres and fibres reinforced with ceramic nanopowders. The obtained results are the same as the theoretical assumptions. As for the refractive index, the lowest value of dielectric transmittance in the range of 1.67 was obtained for the layer made of pure polymer nanofibres of PAN (Figure 10).

**Table 3:** Results of ellipsometry measurements of the real part of the dielectric constant obtained for fibrous layers produced at an electrode distance of 20 cm (the values are provided for a wavelength of 1800 nm).

sample	real part of the dielectric constant

PAN	1.67
PAN/4 wt % TiO_2_	2.16
PAN/8 wt % TiO_2_	2.64
PAN/12 wt % TiO_2_	2.81
PAN/4 wt % Bi_2_O_3_	1.94
PAN/8 wt % Bi_2_O_3_	2.19
PAN/12 wt % Bi_2_O_3_	3.07
PAN/4 wt % SiO_2_	1.71
PAN/8 wt % SiO_2_	1.77
PAN/12 wt % SiO_2_	1.98

The lowest increase in the dielectric constant due to the reinforcing phase was observed for SiO_2_ nanoparticles. Adding an amount of 4 wt % resulted in increase of dielectric transmittance only by 0.04, adding 8 wt % led to an increase of ε by 0.1 compared to the pure polymer. Adding 12 wt % SiO_2_ nanoparticles caused an increase in the dielectric constant value of 1.98. Also for the other particles an increase of the dielectric constant accompanying the addition of the reinforcing phase was observed. Reinforced polymer with 4 wt % TiO_2_ exhibited a dielectric transmittance of 2.16. A further increase of the mass concentration of titanium dioxide nanoparticles led to a significant increase in the dielectric constant to 2.64 (8 wt %) and 2.81 (12 wt %). The maximum value of the dielectric transmittance of the studied fibrous layers was obtained for composite nanofibres reinforced with nanoparticles of Bi_2_O_3_. Adding them to the polymer in an amount of 12 wt % resulted in an almost twofold increase in the dielectric constant relative to the value of the pure polymer, leading a final value of 3.07. Using Bi_2_O_3_ nanoparticles at the lowest mass concentration resulted in a similar result to that obtained in the case of using SiO_2_ nanoparticles at the maximum mass concentration. In this case, the dielectric constant was 1.94. Fibrous layers reinforced with 8 wt % Bi_2_O_3_ exhibited a dielectric transmittance similar to that obtained for fibres reinforced with 4 wt % titanium dioxide, namely 2.19.

The analysis of the relative dielectric transmittance of the produced fibrous mats coincides with the theoretical assumptions arising from the relationship *n* = ε^1/2^ and the obtained results of the refractive index of these materials. With an increase of the mass concentration of the used nanoparticles, there is an increase of the refractive index, and associated with it an increase in the dielectric constant.

#### 3.2 The study of the width of the energy gap of the produced layers

The dependence of the absorption coefficient of a studied material on the energy of the incident radiation on its surface is [40]:

[6]
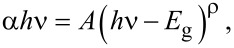


where α is the absorption coefficient, *h* is Planck's constant, ν is the frequency of electromagnetic radiation, *E*_g_ is the the energy gap width and *A* is a constant depending on the probabilities of electron transitions. For the coefficient ρ different values are provided [41], such as 1/2 and 3/2 in the case of accessible and inaccessible direct interband transitions and 2 and 3 for successively permissible and prohibited intermediate transitions. However, the best results were obtained using an exponent equal to 1/2 [42] and this was also done herein.

**Figure 11:**
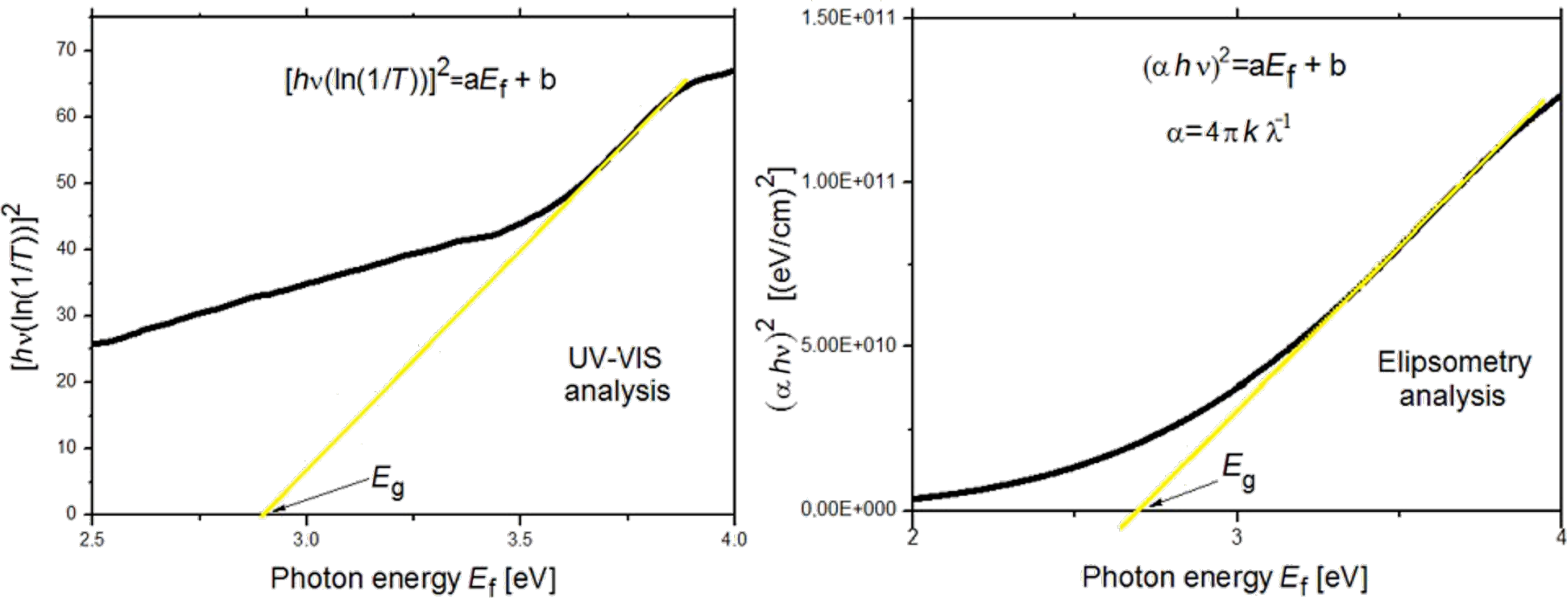
The dependence (α*h*ν)^2^ as a function of the photon energy. Left: the fit obtained from the UV–vis analysis (absorbance vs wavelength). Right: the fit obtained from ellipsometry analysis (extinction as a function of wavelength).

The studies of the energy gap width were carried out with two methods. The first was using ellipsometry studies and provided the values of the extinction coefficient *k*, i.e., the imaginary part of the refractive index. Using the relationship between the absorption coefficient α and the extinction coefficient *k*:

[7]
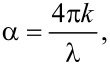


where λ is the length of the electromagnetic wave incident on the sample, and with Equation 6, the relationship [(4π*k*/λ)hν]^2^ was determined as a function of the photon energy. Then, using the method of least squares, a linear function was fitted to the straight parts of the curves, showing the highest slope (Figure 11). The *x*-intercept of the fitted linear functions determines the width of the band gap.

The second method allowing one to determine the relationship between the absorption coefficient α and the energy of the electromagnetic waves is using UV–vis spectral analysis of the obtained fibrous layers. In this case, the nanofibres were directly deposited on microscope slides. For such prepared samples, the absorbance ABS was measured as a function of the wavelength λ. Using the relationship between the absorbance ABS and transmittance T

[8]
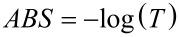


the transmittance as a function of the wavelength was determined. Considering the above, as well as assuming that the reflectivity occurring at the air/sample interface is negligible, Equation 6 takes the following form:

[9]
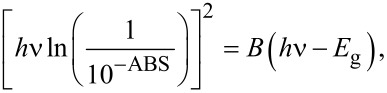


where *B* is a constant depending on the probabilities of electron transitions divided by the thickness of the studied layer. Then, [*h*ν·ln(1/10^−ABS^)]^2^ was determined for all samples, and by analogy with the first method, linear functions were fitted to the straight sections of the curves with the largest slope (Figure 11). The *x*-intercept corresponds to the width of the energy gap of the studied composite fibres. Table 4 shows the results of the studies of the width of the energy gap for the produced fibrous mats, determined using ellipsometry studies and the UV–vis analysis.

**Table 4:** Summary of the determined energy gap widths.

sample	energy gap width [eV]	
	determined from the elipsometry studies	determined from the spectroscopy studies

PAN	2.44	2.57
PAN/4 wt % TiO_2_	2.21	3.08
PAN/8 wt % TiO_2_	2.46	3.15
PAN/12 wt % TiO_2_	2.53	3.19
PAN/4 wt % Bi_2_O_3_	2.51	2.78
PAN/8 wt % Bi_2_O_3_	2.69	2.9
PAN/12 wt % Bi_2_O_3_	2.86	2.99
PAN/4 wt % SiO_2_	2.19	2.59
PAN/8 wt % SiO_2_	2.24	2.74
PAN/12 wt % SiO_2_	2.31	2.89

Each of the used methods showed that the energy gap width increased with increasing mass concentration of the reinforcing phase (Table 4). With the UV–vis spectroscopy measurements, the smallest energy gap width was recorded for nanofibres obtained from pure polymer (2.57 eV, literature data 2.21 eV [43]). In the case of using the extinction coefficients obtained in the ellipsometry studies, the smallest width of the energy gap, smaller than the energy gap determined for pure polymer (2.44 eV), was obtained for composite nanofibres reinforced with nanoparticles of SiO_2_ at all used mass concentrations. While the highest values of *E*_g_ in this method were provided for Bi_2_O_3_-reinforced nanofibres, for which the maximum width of the band gap, amounting to 2.86 eV, was obtained for a mass concentration of 12 wt %. The method using spectroscopy studies showed the highest values of the energy gap width in the case of using TiO_2_ as the reinforcing phase. For all of the used mass concentrations of titanium dioxide particles, the resulting value of the energy gap exceeded 3 eV. The discrepancies in the results obtained using both of these methods can result from using different types of substrates, on which the nanofibres were deposited. In the ellipsometry studies, it was necessary to use silicon wafer as the substrate, and the UV–vis study required the use of nanofibres on the microscope slides. The limitation also involved the very process of producing the fibrous layers, which, due to their complexity, made it impossible to obtain a layer of uniform thickness over their entire surface, which could have affected the measurements.

## Conclusion

PAN nanofibres and composite nanofibres of PAN reinforced with ceramic nanopowders of TiO_2_, Bi_2_O_3_ and SiO_2_, at concentrations of 4, 8 and 12 wt %, were prepared from solution using electrospinning. An analysis of the distance applied between the electrodes (12.5 and 20 cm) revealed that for both the polymer nanofibres without reinforcement, as well as for the composite fibres, the decreased distance between the nozzle and the collector resulted in an increase of the obtained fibre diameters. The concentration of the reinforcing phase and its type also have a significant influence on the morphology of the fibrous layers. With both used distances and for all produced nanocomposites, an increase of the mass concentration of nanoparticles was accompanied by increased diameters of the obtained fibres. Only nanofibres of PAN were devoid of structural defects and showed a constant diameter along their entire length, which was caused by the absence of ceramic nanoparticles as the reinforcing phase. The PAN/TiO_2_ nanofibres showed a tendency for the formation of agglomerates of particles on their surface. In the case of fibres reinforced with Bi_2_O_3_ nanoparticles, structural defects in the form of spindled beads were observed. Most defects in the structure, in the form of surface particle and bead agglomerates, were recorded for composite PAN/SiO_2_ nanofibres. Ellipsometry studies showed that the presence of ceramic nanoparticles in the fibres caused an increase in both the refractive index and the dielectric constant, compared to the values determined for polymer fibres without reinforcement. An increase of the mass concentration of the added nanoparticles was accompanied by an increase in the optical and electrical transmittance, which due to the ability to control the participation of the reinforcing phase, and thus the final dielectric properties of the obtained composites, provides the potential opportunity to use them in the field of electronics. In order to determine the width of the energy gap of the produced fibrous layers, two methods based on the relationship between the absorption coefficient of the studied material and the radiation energy were used. In the first case, the dependence of the extinction coefficient was used in the function of the wavelength, which was determined for each sample during the ellipsometry studies. In this case, the width of the energy gap obtained for pure polyacrylonitrile was 2.44 eV and was lower than the value obtained in the second method, using the analysis of the absorbance, for which the obtained value of the energy gap width was in the range of 2.57 eV. The lowest values of the band gap width, obtained using the ellipsometry analysis, were for PAN/SiO_2_, while the highest values were found for composite fibres of PAN/Bi_2_O_3_. The study of the energy gap, using UV–vis analysis showed that the highest values of *E*_g_, exceeding 3 eV, were obtained for fibres containing particles of TiO_2_, while the lowest increase in the width of the band gap was obtained for a composite of PAN/SiO_2_, as was in the case of studies based on the ellipsometry analysis of the produced samples. The analysis of the electrical properties, using both of these methods, clearly indicated potentially better results by using, as the dielectric material, nanofibres of PAN/TiO_2_ and PAN/Bi_2_O_3_, rather than nanofibres reinforced with SiO_2_ nanoparticles, the determined optical properties of which were similar to the properties obtained for PAN polymer fibre mats. The studies of the band structure of the obtained composite mats indicate that the best dielectric properties were of the layers made of composite PAN nanofibres reinforced with particles of TiO_2_, which proves their attractiveness of application in electronics, inter alia, for the construction of thin-film transistors.
